# Acute and sub-chronic effects of copper on survival, respiratory metabolism, and metal accumulation in *Cambaroides dauricus*

**DOI:** 10.1038/s41598-020-73940-1

**Published:** 2020-10-07

**Authors:** Jie Bao, Yuenan Xing, Chengcheng Feng, Shiyu Kou, Hongbo Jiang, Xiaodong Li

**Affiliations:** Key Laboratory of Livestock Infectious Diseases in Northeast China, Ministry of Education, Key Laboratory of Zoonosis, Shenyang Agricultural University, Shenyang, 110866 China

**Keywords:** Respiration, Ecophysiology

## Abstract

Trace metal contamination in the aquatic ecosystem occurs worldwide: although copper is an essential trace metal, it is considered as a pollutant at certain levels in China. Freshwater crayfish *Cambaroides dauricus* is a commercially important wild species in northeastern China, in which is an important heavy industry area. The population of *C. dauricus* was decreasing sharply due to the environmental pollution and human intervention over the past 20 years. However, nothing is known regarding the responses of this species to trace metal toxicants. This study aimed to determine the acute and chronic toxicity of Cu and its toxicological effects on respiratory metabolism, as well as Cu accumulation in *C. dauricus*. The acute (96 h) median lethal concentration (LC_50_) value of 32.5 mg/L was detected in *C. dauricus*. Then, acute (96 h; 8.24, 16.48 mg/L) and sub-chronic (14 days; 2.06, 4.12 mg/L) exposure in Cu was investigated by estimating the oxygen consumption rate, ammonium excretion rate, and Cu accumulation. Both acute and sub-chronic Cu exposure induced an inhibition of the oxygen consumption rate and ammonium excretion rate, and thereby, an increased O:N ratio. The shift in O:N ratio indicated a metabolic substrate shift towards lipid and carbohydrate metabolism under Cu stress. Cu accumulation in the hepatopancreas and muscles throughout the study was found to be time-dependent and concentration-dependent. The maximum accumulation in the hepatopancreas and muscle were almost 31.6 folds of the control after 14 days’ exposure to 4.12 mg/L concentration. Based on the present work, we suggest that crayfish be considered a potential bioindicator of environmental pollution in freshwater systems. The study provides basic information for further understanding of the toxicological responses of this species to trace metals.

## Introduction

Trace metal contamination in aquatic ecosystems has begun to grow at an alarming rate and now constitutes a significant global problem^1^. As shown in the Report on the State of the Ecology and Environment in China^2^ and the Environmental quality bulletin of China's coastal waters^3^, the high concentration trace metal pollution have been reported in coastal waters, rivers and estuaries, even in the underground water, due to the directly discharged industrial pollution sources, domestic pollution sources and comprehensive drain outlets. For example, copper (Cu) pollution is still the major pollution indicators in marine coastal water and many river systems, which has received considerable attention in China.

Cu is a necessary trace element for the growth and development of all known organisms, including humans and other vertebrates; it mainly acts as a cofactor for various enzymes involved in protecting cells against destruction by oxidation^4^. In crustaceans, copper is a natural component of hemocyanin, the respiratory pigment used in oxygen transport and also plays a role in the molting cycle and in the synthesis of metallothioneins^5^. Owing to the human intervention and industrial activities, Cu discharged from metal mines, smelters, and municipal sewage finds its way into aquatic systems, leading to elevated concentrations in freshwater^6^. For example, the copper concentration of surface water nearby the Dabaoshan Mine of Guangdong province reached up to 10.614 mg/L^7^; the copper level of the rural domestic sewage in Dianchi of Yunnan province was fluctuating between 3.983 to 8.111 mg/L^8^, which were significantly higher than the national water quality standards of China. Moreover, copper sulfate that is traditionally used to control filamentous algae and phytoplankton and external parasites in aquaculture also contributes to the increasing concentration of Cu in aquatic ecosystems^9,10^. As excess Cu is non-biodegradable and persistent, the residue thereby accumulates in the food chain and causes toxicity to aquatic life, even harmful to the human.

The freshwater crayfish, *Cambaroides dauricus*, belonging to Astacidea, Astacoidea, Cambaridae, is widely distributed in China, Russia, and North Korea, and is a commercially important wild species in northeastern China^11^. *C. dauricus* mainly inhabits in shallow waters such as rivers, lakes, and streams with abundant aquatic plants. However, the northeastern China is an important heavy industry area, the serious water pollution, especially of heavy metal pollution, has resulted in the population of *C. dauricus* decreasing significantly to endangered species over the past 20 years. Although many techniques had been used to treat different types of these wastewater that are contaminated with heavy metals such as copper, the seriously damaged ecological environment still cannot be well remedy. The survival aquatic animals in this area appear to exhibit extraordinary tolerance to various environmental contaminants. As far as we know, no research has been examined the effects of trace metals on this species.

The effects of acute and chronic Cu toxicity on crustaceans have been investigated in several studies. Many physiological alterations take place during the exposure period, including metabolic systems, which, in turn, are manifested as growth^10,12^. While the long-time exposure to sub-lethal levels of Cu in crustaceans can further impact their survival, behavior, and reproduction, and eventually change the population quantity^13–17^. Respiration and excretion are the basic physiological activities associated with energy metabolism in animals^18^. They were critical and sensitive indicators to evaluate the physiological responses of environmental conditions^19–21^. Trace metals accumulated in the tissues of the aquatic animals can directly reflect their physiological state. Therefore, the purpose of this investigation was to evaluate the effect of acute (96-h) and sub-lethal chronic (14-d) Cu exposure on respiratory metabolism (manifested in the oxygen consumption rate, OCR; ammonia excretion rate, AER; and O:N) and Cu accumulation in freshwater crayfish, *C. dauricus.* Based on the acute median lethal concentration (LC_50_) values, respiratory metabolism and Cu accumulation were evaluated at concentrations equivalent to 25% (8.24 mg/L) and 50% (16.48 mg/L) LC_50_ for sub-lethal acute assays, and at 6.25% (2.06 mg/L) and 12.5% (4.12 mg/L) LC_50_ for sub-lethal sub-chronic studies. Our data are beneficial to further understand the toxicological responses of *C. dauricus* to heavy metals like Cu.

## Results

### Cu concentration

The measured dissolved Cu concentrations ranged from 91.6 to 98.2% and were close to the normal values (Table 1). Therefore, nominal concentrations of Cu were used for presentation and calculation of toxicity parameters in the tolerance experiments.

**Table 1:** Nominal and measured concentration (mean ± SE, n = 3) of Cu in test solutions.

Normal concentration	Cu concentration (mg/L)						
	0	20	24	29	35	42	50
Measured concentration	0.01	19.63	23.37	28.10	33.73	39.67	45.8
%	–	98.2	97.4	96.9	96.4	94.4	91.6

### Tolerance

The percentage mortality of *C. dauricus* exposed to Cu in each 24-h interval is shown in Table 2. No mortalities were observed in the control. The mortality increased with a corresponding increase in the toxicant concentration at each 24-h interval. Mortality rates of 100% were observed after a 96-h exposure to concentrations of 50 mg/L. The 96-h LC_50_ value was 32.5 mg/L.

**Table 2:** Accumulated mortality (%) of *C. dauricus* exposed to various Cu concentrations for 24, 48, 72, and 96 h and its medium lethal concentration (LC_50_ with 95% confidence limits) calculated by Probit analysis.

Exposure time (h)	Cu concentration (mg/L)							LC_50_ (mg/L)
	0	20	24	29	35	42	50	
24 h	0	0	0	15	40	72	80	39.11 (35.99–42.87)
48 h	0	0	0	27	68	85	90	34.87 (30.23–39.99)
72 h	0	0	0	30	75	85	95	33.80 (29.69–38.24)
96 h	0	0	0	37	77	88	100	32.50 (29.66–35.57)

### Acute metabolic rate responses

Table 3 clearly reveals the inhibition effects on OCR and AER of *C. dauricus* caused by acute Cu exposure. The OCR and AER of *C. dauricus* decreased significantly with increasing exposure to different concentrations of Cu (*P* < 0.05), but there was no significant difference between two exposure concentrations for OCR (*P* > 0.05). After Cu exposure for 96-h, OCR declined by 48.0% at 8.24 mg/L and 48.4% at 16.48 mg/L, while for AER, it decreased by 67.0% and 79.4%, respectively. However, O:N ratios were elevated significantly as Cu exposure concentrations increased (Table 3, *P* < 0.05).

**Table 3:** Effect of acute Cu (96-h) exposure on oxygen consumption rate and ammonia excretion rate in *C. dauricus* (mean ± SE, n = 4).

Exposure concentration (mg/L)	Oxygen consumption rate (mg/g/h)	Ammonia excretion rate (mg/g/h)	O:N ration
Control, 0	0.252 ± 0.018^a^	0.0209 ± 0.0009^a^	10.56 ± 0.57^c^
8.24 (25% 96 h LC_50_)	0.131 ± 0.004^b^ (48.0%)	0.0069 ± 0.0004^ab^ (67.0%)	16.77 ± 1.21^b^
16.48 (50% 96 h LC_50_)	0.130 ± 0.010^b^ (48.4%)	0.0043 ± 0.0001^b^ (79.4%)	27.01 ± 1.76^a^

The values in the parentheses represent percentage decrease over the control. Different letters above the bars indicate significant differences (*P* < 0.05).

### Sub-chronic metabolic rate responses

**Figure 1:**
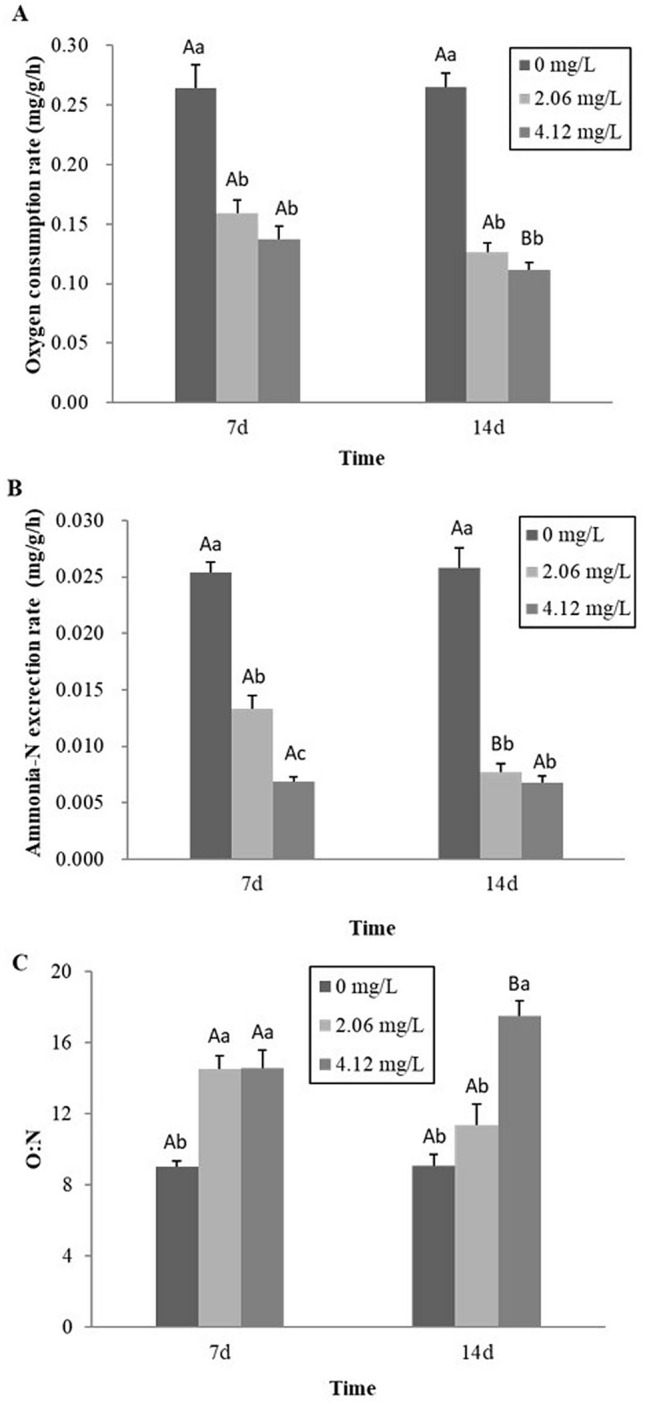
Effects of sub-chronic Cu exposure on oxygen consumption rate (**A**), Ammonia excretion rate (**B**) and O:N ratios (**C**) of *C. dauricus*. The bars are the respective standard errors (n = 4). Note: values with small letters represent significant difference with respect to Cu concentration within a day (*P* < 0.05); Values with big letters mean significant difference with respect to days within a concentration (*P* < 0.05).

Effects of sub-chronic exposure concentration also significantly inhibited the OCR and AER of *C. dauricus* (Fig. 1A,B). At the concentration of 2.06 mg/L, OCR decreased by 39.6% and 52.4% on days 7 and 14 respectively compared to the control group. For the group exposed to 4.12 mg/L Cu, OCR declined by 48.0% and 57.9% on days 7 and 14, respectively (Fig. 1A). However, following exposure to 2.06 mg/L Cu, AER declined by 47.64% and 70.06% at day 7 and day 14, respectively. Additionally, AER decreased by 72.93% and 73.64% following exposure to concentration of 4.12 mg/L (Fig. 1B).

The OCR at day 14 was significantly lower than at day 7 at the highest concentration, but no significant difference was observed at 2.06 mg/L exposure dose (Fig. 1A); while for the AER, significant difference was only detected at 2.06 mg/L exposure concentration (Fig. 1B).

In contrast to its inhibitory effect on OCR and AER, O:N was significantly elevated as the length of exposure concentration, however, exposure time had no significant effect on O:N (Fig. 1C).

### Metal accumulation

**Figure 2:**
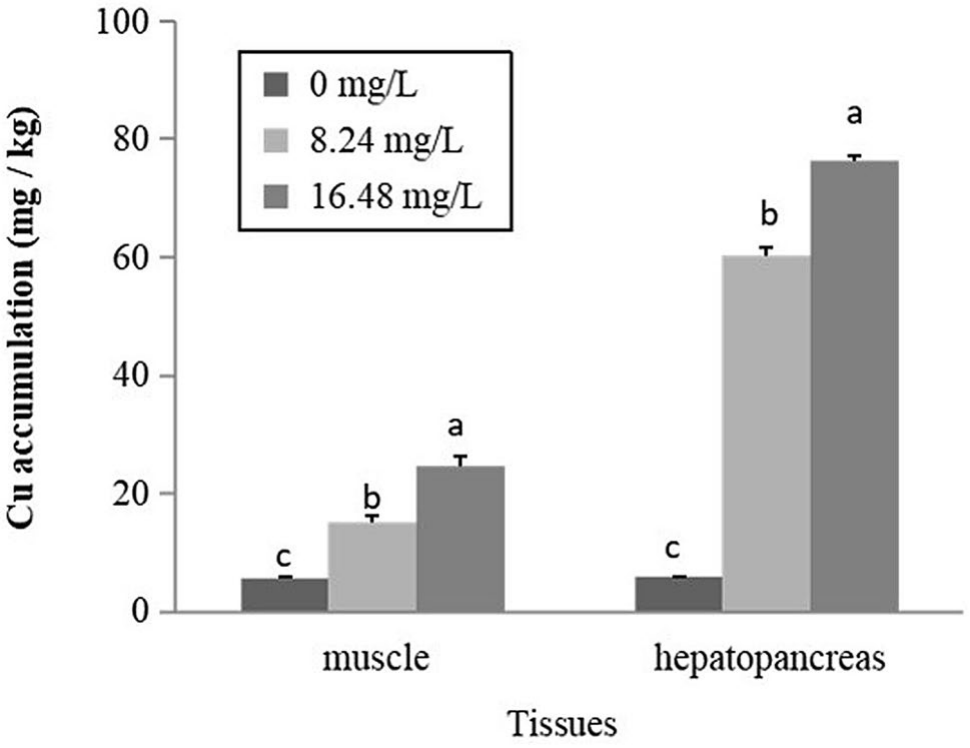
Accumulation of Cu in muscles and hepatopancreas of *C. dauricus* after acute exposure to various concentrations of Cu. Note: The bars are the respective standard errors (n = 4), and different letters above the bars indicate significant differences (*P* < 0.05).

Figure 2 represents the tissue metal concentrations during acute exposure to Cu. The results indicate that there was a significantly gradual increase in the accumulation of Cu in the tissues of muscle and hepatopancreas (*P* < 0.05). At the highest exposure concentration, the Cu accumulation in muscles and hepatopancreas increased by 4.3 folds and 12.7 folds, respectively, compared with the control group.

**Figure 3:**
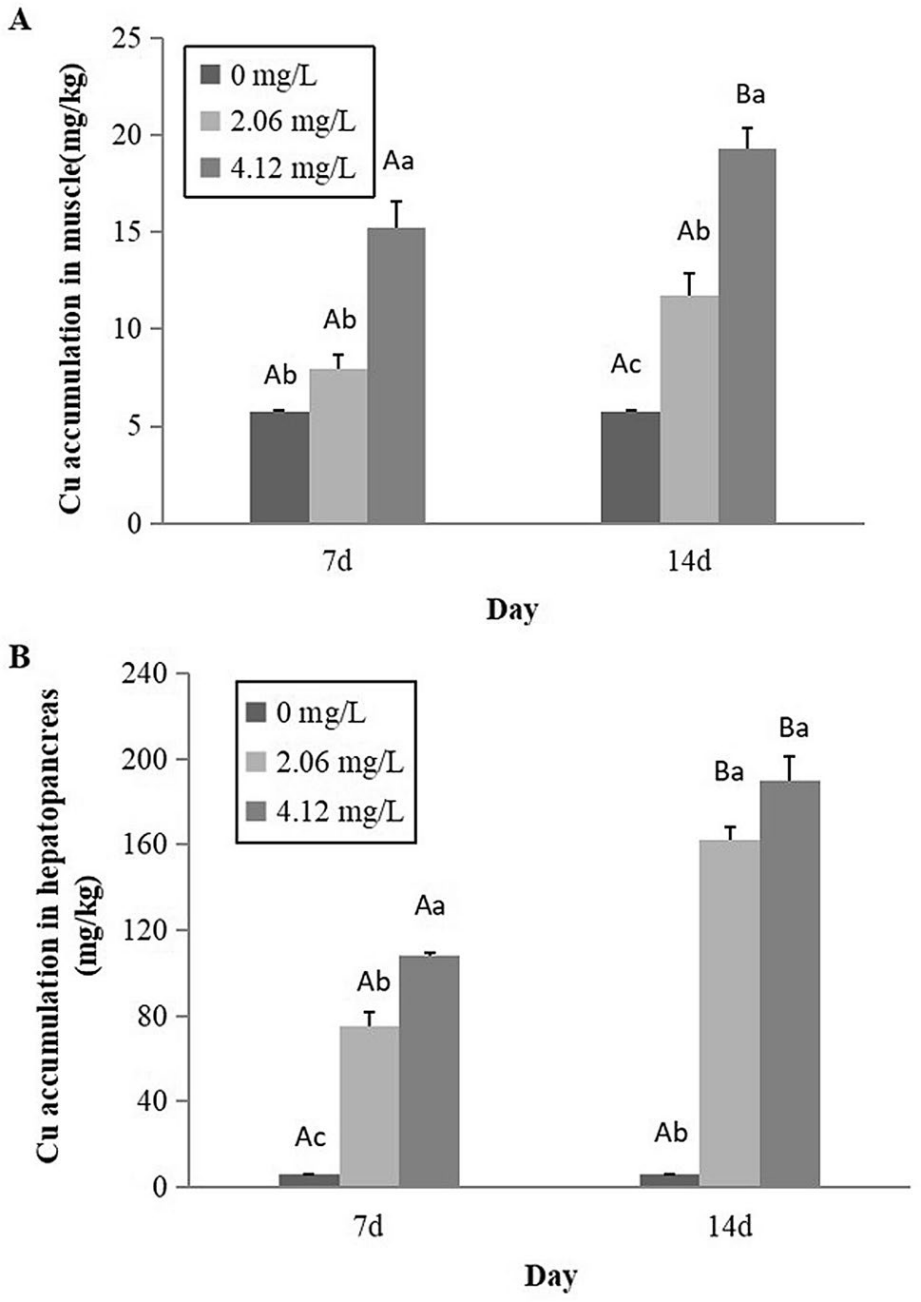
Accumulation of Cu in muscles (**A**) and hepatopancreas (**B**) of *C. dauricus* after sub-chronic exposure to various concentrations of Cu. Note: The bars are the respective standard errors (n = 4). Values with small letters represent significant differences with respect to Cu concentration within a day (*P* < 0.05); Values with big letters represent significant differences with respect to day within a concentration (*P* < 0.05).

Figure 3 represents the metal concentrations in the tissue during sub-chronic exposure to Cu. It was observed that Cu accumulation in muscles and hepatopancreas were significantly affected by various Cu exposure concentrations and time, with longer exposure time and higher exposure concentration leading to higher accumulation (Fig. 3A,B).

In muscle tissues, the significant accumulation concentration was only found at the 4.12 mg/L exposure concentration of 14 days, and it increased by 3.4 folds more than the time-matched control (Fig. 3A). The maximum accumulation in the hepatopancreas was almost 31.6 folds of the control after 14 days’ exposure to 4.12 mg/L concentration (Fig. 3B). When comparation the tissues between hepatopancreas and muscle, the Cu concentration were similar in the control group, but the hepatopancreas accumulated more than 9.8 folds after 14 days exposure at the highest exposure concentration of 4.12 mg/L (Fig. 3A,B).

## Discussion

### Lethal toxicity

Living animals are constantly faced with various environmental stresses that challenge their daily lives. Cu is an essential metal that participates in normal physiological process of crustaceans, but several studies have shown that crustaceans are adversely affected when exposed to high concentrations of Cu. LC_50_ value represents a common point at lethal physiological response to toxicity, which has been well-documented in many crustaceans. For example, the 96-h LC_50_ value for shrimps of *Exopalaemon carinicauda*, *Echinogammars olivii*, *Sphaeroma serratum,* and *Palaemon elegans* was 0.712 mg Cu/L, 0.25 mg Cu/L, 1.98 mg Cu/L, and 2.52 mg Cu/L, respectively^20,22^. In addition, for paddy field crab *Paratelphusa hydrodromus* and freshwater crab, *Barytelphusa cunicularis*, the 96-h LC_50_ values recorded were 15.70 mg Cu/L and 215 mg Cu/L, respectively^23,24^. Likewise, in freshwater crayfish, *Procambarus clarkia,* the 96-h LC_50_ value reached 162 mg Cu/L^25^. These large variations in sub-lethal effects to Cu toxicity in crustaceans appear to be species specific. In our present study, the 96-h LC_50_ value for Cu exposure in *C. dauricus* was 32.5 mg/L, which is much higher than those of the most crustaceans, but this species seems relatively less tolerant to Cu, compared to *P. clarkia*. This difference may also be attributed to the various biotic and abiotic factors like age, sex, weight, salinity, and temperature, besides the species. For example, Taylor^26^ compared the 96-h Cu tolerance of *Cambarus robustus* in Pike Creek and in Wavy Lake and concluded the environment differences could affect population sensitivity to Cu toxicity.

### Oxygen consumption rate

The effects of heavy metal on the respiratory rate of marine and estuarine organisms have been well documented. Spicer and Weber^27^ showed that heavy metal could cause respiratory impairment in crustaceans. The results obtained in the present study confirmed this previous finding. Both acute and sub-chronic Cu exposure induced significant inhibition of OCR in *C. dauricus*, with the maximum decreases of 48.4% and 57.9%, respectively, compared to the control. Similarly, a declined OCR by heavy metal has been observed in shrimps, including *Penaeus indicus*^19^, *L. vannamei*^28^, *F. paulensis*^29^, and *E. carinicauda*^20^, and crabs, including *Uca annulipes*, *U. triangularis*^30^, and *Cancer pagarus*^27^, as well as crayfish, *P. clarkia*^25^. The levels of inhibition of the respiration rate were mainly dependent on the exposure time and exposure concentration. Those authors assumed that the ultrastructural impairments of gill epithelium were related to the decrease in respiration rate, thereby affecting the oxygen carrying capacity of gills. Besides the cytological damage of gill, heavy metals also inhibit mitochondrial energy production, thereby affecting the key metabolic pathways. By contrast, an increased respiration rate has been detected in freshwater shrimp, *Paratya curvirostris*^21^, and lobster *Homarus americanus*^31^. The authors argued that it was attributed to an elevated rate of glycolysis, a mechanism of expenditure of energy reserves characteristic of a stress compensation process. In all, the changes of oxygen consumption level were mainly dependent on the time and concentration of exposure to heavy metals.

### Ammonia excretion rate

Amino acids are the main sources of ammonia production in vivo. Crustaceans have the ability to regulate the concentration of intracellular free amino acids in order to deal with environmental stress^32^. In the present study, AER in either acute or sub-chronic Cu exposure showed a declining trend with increasing exposure concentration and time to Cu. A maximum decrease in AER of 79.4% and 70.06%, respectively, were observed respectively after exposure to 16.48 mg/L for 96-h and 2.06 mg/L for 14 days, in comparison to the control (Fig. 1B). In a similar manner, Chinni^19^ also reported a significant decrease in AER in post larvae *P. indicus* when exposed to Pb for 30 days. It assumed that such a decrease may be due to reduction in the metabolic rate or an interaction of heavy metal with the pathways for the production of ammonia-N. By contrast, elevations of ammonia excretion in response to heavy metals exposure were reported in other crustaceans. For example, an increase in AER was found in juvenile *E. carinicauda* after exposure to Zn and Hg^20^ and in *F. paulensis* after exposure to Cd and Zn^29^. It was considered that the gill function was impaired by the metal exposure, resulting in the dysfunction of ammonium excretion control; therefore, outflow of ammonia excretion from the hemolymph to ambient water induced an increased ammonia concentration in the water. In addition, no change in ammonia excretion rate was obtained in *Paratya curvirostris* after 96-h acute and 10-day sub-chronic Cd stress^21^. Therefore, the questions of the relationship between heavy metal exposure and ammonia excretion needs to be properly investigated.

### Energy metabolism

O:N is a useful value for evaluating the characteristics of nutrients utilized by animals and can provide information on changes in energy substrate utilization under various environmental stresses^33,34^. Theoretically, pure protein catabolism will produce an O:N ratio of 8^35^, and equal proportions of proteins and lipid results in an O:N of 24^36^. An O:N ratio higher than 24 indicates an elevation in lipid and carbohydrate metabolisms. In this study, in comparison with the controls, high values of O:N were obtained in individuals of *C. dauricus* exposed to Cu for 96 h and 14 days (Table 1, Fig. 1C). In generally, protein catabolism for energy is less efficient than lipid/carbohydrate catabolism. A species that relies on lipid and carbohydrate metabolism will likely be able to better meet energy demands of toxicant exposure than a species that principally metabolizes protein. The mean O:N ratio higher than 24 in acute Cu exposure and lower than 24 in sub-chronic exposure (Table 3 and Fig. 1C) indicated the differences in energy utilization strategy in response to two patterns of Cu stress. This could be a mechanism explaining the differences in energetic responses to Cu exposure in *C. dauricus*, relative to other crustacean species.

### Tissues accumulation

Cu is an essential trace element for biological processes, particularly as a component of the respiratory pigment, hemocyanin. The body Cu concentration in decapod crustaceans can be regulated and does not accumulate until certain environmental threshold levels are achieved^37^. In addition, as an economic species of crustaceans and in relation to food quality and safety assessment, organ-specific accumulation data, especially for the muscle, are markedly required. In this study, tissue-specific bioaccumulation of Cu observed, and the Cu accumulation in hepatopancreas and muscles were highly dependent on water Cu concentration and exposure time (Fig. 2; Fig. 3A, 3B). Hepatopancreas is the organ most associated with the detoxification and biotransformation process and in direct contact with toxicants in water. The hepatopancreas, containing metal-binding protein, is the main target organ for regulating Cu level^38^. The maximum Cu accumulation was observed in hepatopancreas, which increased 12.7 folds and 31.6 folds after 4-day acute exposure to 16.48 mg Cu/L and chronic 14-day exposure to 4.12 mg Cu/L, respectively, this indicated that *C. dauricus* had a great potential for rapid accumulation of Cu in fresh waters. The greatest Cu accumulation occurring in hepatopancreas had been reported for the crayfish species, *Astacus leptodactylus*^39^ and *Procambarus* sp.^40^ as well as for the freshwater prawn, *M. rosenbergii*^38^. Although the hepatopancreas could regulate the Cu level in the animal’s body to avoid toxicity and deficiencies, the high level of external water Cu breaks down the regulation of Cu and causes continuous Cu accumulation, which might lead to the loss of muscular control and eventually, death, for crustaceans.

In this study, there was no significant time-dependent trend in the accumulation of Cu in the muscle between 7 and 14 days of Cu stress in the lower concentration of 2.06 mg/L (Fig. 3A), this suggests that *C. dauricus* was able to regulate Cu in the muscle to a fairly constant level under low Cu exposure concentrations. However, *C. dauricus* exposed to concentration of 4.12 mg Cu/L showed increased accumulation of Cu in the muscle and the equilibrium of Cu accumulation was not reached at 14 days, which might show that the high level of Cu in the external water breaks down the regulation of Cu and caused a continuous Cu accumulation, leading to its toxicity at high concentration. Similar result had been reported in *Procambarus* sp.^40^. The author found that Cu uptake reached a kinetic equilibrium within 10 days of exposure to 0.31 mg Cu/L in five organs (gills, ovaries, exoskeleton, hepatopancreas, and muscles), but Cu was rapidly accumulated in the organs of most *Procambarus* sp., especially in the hepatopancreas, when exposed to higher concentration of 0.38 mg Cu/L after the 14-d exposure test. However, muscle tissue, as the main edible portion, accumulates Cu at a relatively lower rate (Fig. 2; Fig. 3A) and this is important from the angle of human food quality and safety.

### Conclusion

In this study, we observed that the acute and sub-chronic toxicity of Cu had a dramatic impact on the survival, oxygen consumption rate, ammonia excretion rate and bioaccumulation of *C. dauricus*. *C. dauricus* mainly took the strategies of inhibiting respiratory metabolism and shifting energy utilization to adapt to copper stress. The *C. dauricus* had higher concentration-dependent accumulation ability of copper. Our future work will focus on the metabolic characteristics of copper and other heavy metal from the angle of human food safety. Therefore, our studies provided basic information for further understanding of the toxicological responses of this species to trace metals.

## Methods

### Experimental animals and rearing conditions

Juvenile *C. dauricus* were collected from a culture farm at Benxi, Liaoning, PR China. The crayfish were transferred to the laboratory and acclimated in tanks (250 L water) for 15 days. During the period of acclimation and experiment, crayfish were fed formulated diet (crude protein 36%) to satiation once a day. A photoperiod of 12 h light:12 h dark was controlled and natural water temperature was at maintained at 24 ± 1 °C. Filtered recycling water was replaced every day and aeration was provided continuously. Intermolt crayfish were selected and used as experimental animals.

### Tolerance test

The six Cu concentrations tested were 20 mg/L, 24 mg/L, 29 mg/L, 35 mg/L, 42 mg/L, and 50 mg/L. The 100 g/L stock solutions of Cu were freshly prepared by dissolving CuSO_4_·5H_2_O (analytical grade, Sinopharm chemical reagent Co. Ltd, Shanghai, China), and the stock solutions were diluted to the tested concentration by adding the calculated volume of stock solutions to 50 L water in a 67.5 L (45 × 25 × 60 cm) glass aquarium. A control treatment without metal toxicant was maintained. Each Cu test concentration treatment was carried out in three replicates, with each aquarium holding 20 individuals. A total of 420 individuals with an initial bodyweight of 8.5 ± 0.5 g (S.E.) were randomly selected from the acclimated animals and stocked into 21 glass aquaria, including the control. Acute Cu exposure experiment lasted for 96 h. The tested crayfish were fasted during this period. All test solutions were replaced at 24 h to maintain the test metal concentration. Dead crayfish were removed, and mortality was recorded daily.

### Metabolic experiments

To determine the effect of acute (96-h) sub-lethal Cu on OCR and AER of *C. dauricus*, final doses of 8.24 mg/L and 16.48 mg/L were set. These corresponded to normal exposure concentrations of 25% and 50% 96-h LC_50_ values, respectively. To evaluate the sub-lethal chronic (14 d) effects on *C. dauricus*, concentrations equivalent to 6.25% (2.06 mg/L) and 12.5% (4.12 mg/L) 96-h LC_50_ values were used. As the higher tolerance of *C. dauricus* to the Cu stress, the acute and chronic experimental Cu concentration were set relative higher to acquire the stress effects. The experimental Cu concentration of chronic stress was set close to the safe concentration. For acute and sub-chronic exposure studies, acclimated crayfish were randomly selected and 10 individuals each were stocked in 67.5 L glass aquaria (45 × 25 × 60 cm). Prior to the renewal of test solutions at 24 h, crayfish were fed formulated diet and allowed to feed for 1 h; then half of the tested water was replaced every day to maintain constant concentration of Cu. The exposure was conducted under 12 h light:12 h dark cycle, at temperature of 24 ± 1 °C.

Improved closed-bottle respirometry methods were employed to determine OCR and AER^41^. The crayfish were fasted for 24 h before estimating the metabolic rates. At 96 h, or Day 7 and Day 14, two individuals were selected and placed in 2 L respiration chambers, with 4 replicates (chambers) per treatment. Crayfish were carefully placed in each chamber and allowed to acclimate for 2 h attenuate the handling stress. Then water gently flowed through the chamber until it overflowed the rims more than three times, after which the chamber was sealed immediately. Four blank chambers served as control for each Cu concentration. The experiment lasted for 2 h and the water dissolved oxygen at the end of experiment was maintained above 4.0 mg/L to avoid physiological stress. Then, water samples from each chamber were collected for the determination of OCR and AER; the concentrations were estimated by Winkler and indophenol blue methods, respectively.

### Cu accumulation

For acute exposure and sub-chronic exposure experiments, *C. dauricus* were exposed to the same Cu concentration as that in the metabolic experiments (8.24 mg/L and 16.48 mg/L for acute exposure experiment, 2.06 mg/L and 4.12 mg/L for sub-chronic exposure experiment). At 96 h, or days 7 and 14, the hepatopancreas and muscle tissues of four individuals in each treatment were collected, weighed, and frozen at − 80 °C for further Cu estimation. The frozen tissues were lyophilized and digested at digested in 5.0 ml concentrated HNO_3_ for 2 h at 120 °C. Then, 1.0 ml H_2_O_2_ was added and boiled for 1 h. The digest was then diluted to 20 mL using Milli-Qwater and tissue Cu was determined by atomic absorption spectrophotometry in the flame mode (Z2000, Hitachi, Japan). Quality control was achieved by using certified reference material SRM 2976 (National Institute of Standards and Technology, US), with a recovery rate of between 92 and 105%. Data for tissue samples were expressed as mg/kg tissue.

### Data calculation and statistical analysis

The OCR, AER, and oxygen consumed to nitrogen ratio (O:N) were calculated with the following equation:

$$ \begin{aligned} & {\text{OCR }}\left( {{\text{mg}}/{\text{g}}/{\text{h}}} \right) = \left( {DO_{C} - DO_{E} } \right) \times V/\left( {W \times t} \right) \\ & {\text{AER }}\left( {{\text{mg}}/{\text{g}}/{\text{h}}} \right) = \left( {{\text{A}}E_{E} {-}{\text{A}}E_{C} } \right) \times V/\left( {W \times t} \right) \\ & {\text{O}}:{\text{N ratio}} = \left( {{14 } \times {\text{OCR}}} \right)/\left( {{16} \times {\text{AER}}} \right) \\ \end{aligned} $$

where *DO*_*C*_ and *DO*_*E*_ are oxygen concentrations in the control and experimental chambers (mg/L), respectively; *AE*_*C*_ and *AN*_*E*_ are concentrations of excreted ammonia in the control and experimental chambers (mg/L), respectively; *V* is the volume of the respiration chamber (L); *W* is the wet body weight of the shrimp (g); *t* is the metabolic duration (h).

Data analyses were performed with SPSS 16.0 for Windows. The lethal concentrations of different Cu solutions and the 95% confidence intervals were calculated by Probit analysis. All the data were checked for variance homogeneity by Levene's test and for distribution normality by Shapiro–Wilk's test. Significant differences were assessed by one-way or two-way ANOVA (with time and concentration as the two factors) followed by the post-hoc Tukey test if the conditions were met, or with non-parametric tests: Kruskal Wallis. *P* < 0.05 was considered significant, and all data are presented as means ± SE.
